# EQUIP: Implementing chronic care principles and applying formative evaluation methods to improve care for schizophrenia: QUERI Series

**DOI:** 10.1186/1748-5908-3-9

**Published:** 2008-02-15

**Authors:** Alison H Brown, Amy N Cohen, Matthew J Chinman, Christopher Kessler, Alexander S Young

**Affiliations:** 1 VA Desert Pacific Mental Illness Research, Education, and Clinical Center, Los Angeles, California, USA; 2 Veterans Affairs Greater Los Angeles Healthcare System, Los Angeles, California, USA; 3 UCLA Department of Psychiatry, Los Angeles, California, USA; 4 RAND Corporation, Santa Monica, California, USA

## Abstract

**Background:**

This paper presents a case study that demonstrates the evolution of a project entitled "Enhancing QUality-of-care In Psychosis" (EQUIP) that began approximately when the U.S. Department of Veterans Affairs' Quality Enhancement Research Initiative (QUERI), and implementation science were emerging. EQUIP developed methods and tools to implement chronic illness care principles in the treatment of schizophrenia, and evaluated this implementation using a small-scale controlled trial. The next iteration of the project, EQUIP-2, was further informed by implementation science and the use of QUERI tools.

**Methods:**

This paper reports the background, development, results and implications of EQUIP, and also describes ongoing work in the second phase of the project (EQUIP-2). The EQUIP intervention uses implementation strategies and tools to increase the adoption and implementation of chronic illness care principles. In EQUIP-2, these strategies and tools are conceptually grounded in a stages-of-change model, and include clinical and delivery system interventions and adoption/implementation tools. Formative evaluation occurs in conjunction with the intervention, and includes developmental, progress-focused, implementation-focused, and interpretive evaluation.

**Results:**

Evaluation of EQUIP provided an understanding of quality gaps *and *how to address related problems in schizophrenia. EQUIP showed that solutions to quality problems in schizophrenia differ by treatment domain and are exacerbated by a lack of awareness of evidence-based practices. EQUIP also showed that improving care requires creating resources for physicians to help them easily implement practice changes, plus intensive education as well as product champions who help physicians use these resources. Organizational changes, such as the addition of care managers and informatics systems, were shown to help physicians with identifying problems, making referrals, and monitoring follow-up. In EQUIP-2, which is currently in progress, these initial findings were used to develop a more comprehensive approach to implementing and evaluating the chronic illness care model.

**Discussion:**

In QUERI, small-scale projects contribute to the development and enhancement of hands-on, action-oriented service-directed projects that are grounded in current implementation science. This project supports the concept that QUERI tools can be useful in implementing complex care models oriented toward evidence-based improvement of clinical care.

## Background

Shortly after the inauguration of the U.S. Department of Veterans Affairs (VA) Quality Enhancement Research Initiative (QUERI) in 1998, a Request for Proposals (RFP) was released for Investigator-Initiated Research (IIR) projects that focused on implementing clinical guidelines in VA healthcare facilities. Recognizing that implementation of guidelines was not a straightforward endeavor, the RFP suggested particular attention be paid to *barriers to guideline implementation*, such as "provider issues (knowledge, attitudes, and behavior) and system issues (e.g., resources, culture, patient population, etc.)." At that time, "implementation" was to be operationalized "in terms of observed changes in practice and, when possible, changes in patient and system outcomes (cost, quality of care, average length of stay, policy or procedure changes, practice variations), i.e., not mere dissemination of, or pronouncements about, guidelines." The science of implementation was still in development; specific methods for engaging in implementation science had not yet been spelled out, and instead, more traditional approaches were being used to design and assess the process of implementation.

In this paper, we present the evolution of a project that began approximately when QUERI and implementation science began, and that has been transformed, with continued funding, into a project that explicitly engages in implementation science as it is currently defined and operationalized within QUERI [1]. The initial project, "Enhancing QUality-of-care In Psychosis," or EQUIP, developed methods and tools to apply a chronic illness care model in schizophrenia, and evaluated the implementation of this care model using a small-scale controlled trial. The EQUIP intervention used strategic tools to increase the adoption and improve the implementation of this care model. It included substantial qualitative methods, though its formative evaluation was modest by current standards.

Evaluation of EQUIP led to a more recent project, EQUIP-2, which is a larger-scale trial of the chronic illness care model implementation currently in progress. Tools from EQUIP have been refined, and improvements have been made to the original implementation method. In addition, EQUIP-2 incorporates a more complete formative evaluation to optimize future, broader implementation of the EQUIP intervention. Our ability to design the project in this way reflects recent advances that have been made in the science of implementation, particularly with regard to the various types of formative evaluation that can be used over the stages of a project [2]. In describing the evolution of the EQUIP project, we illustrate the value of the QUERI expectation that study development and refinement should occur in implementation research within and across phased, improvement-focused projects. We hope the paper will stimulate additional scientific discussion about the challenges of implementation.

This article is one in a *Series *of articles documenting implementation science frameworks and approaches developed by the U.S. Department of Veterans Affairs (VA) Quality Enhancement Research Initiative (QUERI). QUERI is briefly outlined in Table 1 and described in more detail in previous publications [3,4]. The *Series' *introductory article [1] highlights aspects of QUERI that are related specifically to implementation science, and describes additional types of articles contained in the *QUERI Series*.

**Table 1:** The VA Quality Enhancement Research Initiative (QUERI)

The U.S. Department of Veterans Affairs' (VA) Quality Enhancement Research Initiative (QUERI) was launched in 1998. QUERI was designed to harness VA's health services research expertise and resources in an ongoing system-wide effort to improve the performance of the VA healthcare system and, thus, quality of care for veterans.
QUERI researchers collaborate with VA policy and practice leaders, clinicians, and operations staff to implement appropriate evidence-based practices into routine clinical care. They work within distinct disease- or condition-specific QUERI Centers and utilize a standard six-step process:
1) Identify high-risk/high-volume diseases or problems.
2) Identify best practices.
3) Define existing practice patterns and outcomes across the VA and current variation from best practices.
4) Identify and implement interventions to promote best practices.
5) Document that best practices improve outcomes.
6) Document that outcomes are associated with improved health-related quality of life.
Within Step 4, QUERI implementation efforts generally follow a sequence of four phases to enable the refinement and spread of effective and sustainable implementation programs across multiple VA medical centers and clinics. The phases include:
1) Single site pilot,
2) Small scale, multi-site implementation trial,
3) Large scale, multi-region implementation trial, and
4) System-wide rollout.
Researchers employ additional QUERI frameworks and tools, as highlighted in this *Series*, to enhance achievement of each project's quality improvement and implementation science goals.

Below we provide a brief overview of the Mental Health QUERI Center which supports the current project. We then briefly describe EQUIP and the evaluation research methods used in the project, followed by a presentation of the EQUIP findings. The second half of the paper concentrates on a description of EQUIP-2 methods, which was funded through a different mechanism than EQUIP, and, as noted above, is more clearly a project that engages in implementation science. We conclude by reflecting on the utility of the QUERI process and proposing directions for future hands-on, action-oriented research [4].

### The Mental Health QUERI (MHQ) focus on schizophrenia

Schizophrenia is a chronic medical disorder that occurs in about 1% of the population, and results in substantial morbidity and mortality when poorly treated. Although evidence-based practices (EBPs) improve outcomes in schizophrenia, these treatments are not often used [5,6]. Some EBPs, such as 'Assertive Community Treatment' and 'Individualized Placement and Support,' have not been widely implemented, thereby limiting patient access. For other EBPs, such as clozapine (commonly used drug for schizophrenia) and caregiver services, clinicians may lack the competencies to deliver them, and typical clinic organization is not consistent with their use. In addition, evidence-based quality improvement (EBQI, [7]) has been nearly impossible in treating schizophrenia because existing medical records (including electronic medical records) lack reliable information regarding patient symptoms, side-effects, and functioning [8]. Although national organizations, including the VA, have made implementation of appropriate care for schizophrenia a high priority [9,10], there has been only modest success in developing interventions to overcome implementation barriers [11-13]. Clearly, interventions that enhance the implementation of evidence-based treatments are needed in schizophrenia. The interventions tested in EQUIP and EQUIP-2 involve tools for supporting the implementation of chronic illness care principles in schizophrenia.

## EQUIP Methods

This section describes the EQUIP project and implementation intervention methods in more detail, and the methods used in the formative evaluation component of EQUIP.

### EQUIP overview and specific aims

Funded in 2001, the goals of EQUIP were to develop, implement and evaluate a strategy designed to apply the chronic illness care model to the outpatient treatment of schizophrenia in a Step 4, Phase 1 QUERI project (see Table 1). As noted above, projects responding to the 1998 RFP were geared toward "guideline implementation." In schizophrenia, application of a chronic illness care model requires attention to several sets of pertinent guidelines, including established principles of chronic illness management [14,15] and national treatment guidelines for schizophrenia. At the time of EQUIP, these guidelines included the American Psychiatric Association guidelines [16], the Agency for Healthcare Research and Quality Patient Outcomes Research Team (PORT) treatment recommendations [17], and a VA treatment algorithm (These guidelines have subsequently been updated [18,19]). Taken together, the EQUIP care model focused on improving treatment in three domains: 1) treatment assertiveness and care coordination, 2) guideline-concordant medication management of symptoms and side-effects, and 3) family services.

The specific aims of EQUIP were to: 1) Assess, in a randomized, controlled trial, the effect of a chronic illness care model for schizophrenia relative to usual care on: a) clinician attitudes regarding controlling symptoms and side-effects, and regarding family/caregiver involvement in care; b) clinician practice patterns and adherence to guideline recommendations; c) patient compliance with treatment recommendations; d) patient clinical outcomes (e.g., symptoms, side-effects, quality of life, and satisfaction); and e) patient utilization of treatment services; and 2) Assess, using mixed qualitative and quantitative methods, the success of the implementation strategy's impact on uptake of the model.

### EQUIP research design and methods

The chronic illness care principles were evaluated at two outpatient mental health clinics within two large, urban VA medical centers in Southern California. At these two clinics, psychiatrists were randomized to the best practice intervention (care model) or control (treatment as usual). Case managers and patients were assigned to the same study arm as the psychiatrists with whom they were associated. At the third clinic, within one of the medical centers, all the clinicians and patients were assigned to the control group. The chronic illness care model intervention was developed, implemented and fully operational in January 2003 and was sustained for more than 15 months. The relevant institutional review boards approved all trial procedures.

Clinicians were eligible for the study if they practiced at one of the clinics. Eligible clinicians were given information about the study and the opportunity to enroll. Patients were eligible if they were at least 18 years old, had a diagnosis of schizophrenia or schizoaffective disorder, had at least one visit with an enrolled psychiatrist during a four-month sampling period immediately before the enrollment period (i.e., "visit-based sampling" [6]), and had at least one clinic visit during a five-month enrollment period. When an eligible patient came into the clinic during the enrollment period, he or she was provided with information about the study and was given the opportunity to enroll. The intervention included 32 psychiatrists, 1 nurse practitioner, 3 nurse case managers, and 173 patients. The control group included 43 psychiatrists, 1 psychiatric pharmacist, 3 nurse case managers, and 225 patients. Informed consent was obtained from all patients, or their legal conservators, and all clinicians.

The study included clinical interventions, delivery system interventions, and adoption/implementation tools. These interventions and tools are presented in Table 2. One of the innovations of EQUIP was the use of the Medical Informatics Network Tool (MINT) to provide instant, summarized clinical information (via a "PopUp" window, see Table 2) to clinicians as they accessed the patient's medical record. As noted in the table, adoption and implementation tools also were utilized to enhance the utility and effectiveness of the intervention.

**Table 2:** EQUIP intervention components

Clinical intervention
• Chronic illness care model aimed at lessening psychotic symptoms and medication side effects and increasing family/caregiver involvement in care
Delivery system interventions
• Research nurse (RN) stationed at each of the clinics assessed every intervention patient at each visit.
• Protocols for assertive, coordinated care.
• Resources supporting evidence-based medication management and family services [37].
• "Medical Informatics Network Tool" (MINT, [21]), an informatics system that collected and managed outcomes data in real time and worked in conjunction with the VA's fully electronic medical record.
• MINT generated a window ("PopUp") each time an enrolled provider opened the electronic medical record of an intervention patient.
• The PopUp window contained the RN's clinical assessment, with urgent issues highlighted. The PopUp provided links to treatment guidelines, and allowed for secure messaging among the clinical team members.
• MINT produced Quality Reports to track data regarding the clinical status of the psychiatrist's patients in three domains: compliance and caregiver problems, symptoms, and medication side-effects.
• Quality Reports were distributed quarterly by the research nurse to enrolled psychiatrists.
Adoption/implementation tools
• Marketing of the care model via educational activities and trainings.
• Partnerships with clinic personnel.
• Product champions were nominated by the site PI mid-intervention. They were asked to promote the goals of the project during regular staff meetings.

### EQUIP formative evaluation methods

In addition to the implementation strategy described above, EQUIP involved formative evaluation. Table 3 depicts the methods that were utilized in the evaluation.

**Table 3:** EQUIP formative evaluation methods

Pre-implementation
• Semi-structured interviews conducted by research personnel with intervention and control psychiatrist participants (n = 35): gathered data on psychiatrists' previous experience with research, their clinical practices, and expected barriers and facilitators of intervention components.
• Self-report questionnaire completed by intervention and control psychiatrist participants (n = 44): gathered data on psychiatrists' training, attitudes, knowledge and skills related to schizophrenia and guidelines, and on workload.
Mid-implementation
• Semi-structured qualitative interviews conducted by an independent contractor with a sub-sample of the intervention clinical and research staff (n = 18): gathered data on usefulness of the PopUps, in order to make any necessary changes that would enhance the remainder of the EQUIP intervention.
• Computer System Usability Questionnaire [38] completed by sub-sample of intervention psychiatrist participants (n = 16): gathered quantitative data on experiences with the PopUps.
• Quality Report survey completed by sub-sample of intervention psychiatrist participants (n = 8): provided data on uptake of the Quality Report they received quarterly.
Post-implementation
• Semi-structured qualitative interviews conducted by research personnel with intervention and control clinical and research staff (n = 11): gathered data on psychiatrists' current clinical practices, barriers and facilitators of intervention components, especially the unsuccessful family component, satisfaction/dissatisfaction with the implementation program, and recommendations for future programs.
• Self-report questionnaire completed by intervention and control psychiatrist participants (n = 14): gathered data on psychiatrists' attitudes, knowledge and skills related to schizophrenia and guidelines, their attitudes about recovery and family services, and on workload.

Pre- and post-implementation semi-structured interviews and surveys were conducted to assess experience with research, clinical practice and competencies, plus expectations and observations of the implementation. At the time of the post-implementation survey, the research team was already planning EQUIP-2 (described below) and, therefore, specific feedback was desired for the next phase of implementation. These pre- *and *post-implementation interviews and surveys were conducted by research staff. There was an attempt to interview and survey as many psychiatrists as possible from both the intervention and control arms of the study.

Mid-implementation interviews and surveys were conducted to assess the process of intervention implementation. These mid-intervention interviews were conducted by an independent contractor (A. Brown). Via surveys, clinicians were asked specifically how the informatics system was working for them, *as well as about *the effect of the Quality Report. Both the mid-implementation interview and survey were conducted with a sub-sample of psychiatrists- those who were most involved with the implementation due to higher caseloads of patients in the sample. As a result of the timing of this mid-implementation feedback, changes were made midway through, to make relevant interventions more effective and appealing.

## EQUIP results

### Main evaluation findings

The evaluation of EQUIP provided an understanding of quality gaps *and *how to address related problems in schizophrenia. Our findings are summarized in the left column of Table 4.

**Table 4:** Findings in EQUIP and resulting adjustments made in EQUIP-2

**EQUIP Finding**	**EQUIP-2 Adjustment: Intervention**
*Clinical interventions*	
Care targets were equally applied at all sites.	Sites choose their preferred care targets based on local needs and resources.
*Delivery system interventions*	
Providers made limited use of symptom assessments performed by highly trained nurse assessors, and questioned the accuracy of the assessments.	Patients complete self-assessments, which are given to relevant providers.
Providers at the clinics had high levels of depersonalization, high levels of exhaustion, and a low sense of personal accomplishment (burn-out).	One clinic staff member included in project calls and meetings in order to modify the care model to local needs and organization. Staff provided with more feedback throughout implementation, including material and other reinforcements for high achievers.
The Quality Report was distributed quarterly by the nurse to each individual psychiatrist, with only modest discussion.	The Quality Report is distributed at monthly staff meetings by the product champion. Quality of care outliers (good and bad) and clinic-wide problems are discussed among the team.
The PopUp included links to summaries of treatment guidelines, but psychiatrists did not use these links.	Treatment recommendations will be "pushed" to psychiatrists in the context of specific patients, and computers will provide patients with education about guideline-concordant treatments.
*Adoption/implementation tools*	
A non-systematic approach to site inception may have affected buy-in and enthusiasm.	A project "kick-off" is highlighted with participation of all sites.
Engagement was primarily with clinic-level personnel.	Engagement occurs with clinic-level personnel, medical center personnel, and regional policy-makers.
Case managers were important, but were available only at one site and entered the project late.	Case managers are involved from the beginning.
Product champions were appointed by medical center administration late, and were less intensively involved than desired.	Product champions self-identify prior to implementation and are more fully utilized.

EQUIP revealed that solutions to quality problems in schizophrenia differ by treatment domain. For example, challenges to implementing family services proved to be very different from challenges to implementing weight management using wellness groups. Improving family services required assessment of each patient-caregiver relationship, intensive negotiation with patients and caregivers, major care reorganization to accommodate family involvement, and attention to clinician competencies (e.g., knowledge, attitude, and skills). Improving weight and wellness required assessment of the problem in each patient, the establishment of therapeutic groups, involvement of nutrition and recreational services, and help with referrals and follow-ups.

EQUIP also revealed that quality problems can arise from poor clinician competencies [20]. For example, we found clinician competency problems in the use of clozapine. This clozapine competency problem is well established anecdotally, although there is little empirical evidence of it. The main competency problems that we encountered, in at least a subset of clinicians, were: 1) clinicians were not trained in the use of clozapine, or had not used it despite training; 2) clinicians were not credentialed to use clozapine in their settings; 3) clinicians were discouraged by the possibility that having patients on clozapine would necessitate longer clinical visits with more clinical effort; and/or 4) clinicians did not believe clozapine would be helpful. Quality problems can also arise due to difficulty in changing psychiatric treatments. In EQUIP, we noted that psychiatrists made minimal use of data showing that their patients had high levels of symptoms and side-effects (Quality Reports), and they also made minimal use of the guidelines that were easily accessible via the MINT "PopUp" (see Table 2) that was available on their computer at every clinical encounter [21].

Quality problems such as these can be exacerbated by a general lack of awareness of evidence-based practices, such as approaches to managing increased weight or treatment-refractory psychosis. During the course of implementation, it became apparent to the research team that increasing the intensity of follow-up (e.g., adding clinic visits) for severely ill patients was of limited use. Clinicians typically did not change treatments in response to clinical data. Therefore, additional treatment visits were of limited value because they were not likely to lead to appropriate changes in treatment in response to psychosis or medication side-effects.

Based on what we were seeing in terms of these persistent quality problems, we began to conclude that improving care required creating resources to support clinicians and reorganizing care to help them easily implement changes in their clinical practices. Also, there was a need for intensive education and product champions who would work with clinicians to encourage awareness and use of these resources. Care managers and the informatics system did help physicians identify clinical problems in their patients, but these interventions and tools needed reassessment and possibly redesign. For example, we learned, through the involvement of the case managers, that tools designed for clinicians may not have the same appeal across types of clinicians. We found that psychiatrists and case managers (though the sample was small) had differing perspectives on the value of being provided with clinical data by their computer (e.g., PopUps) during the encounter. This supported the assertion that formative evaluation data must be gathered from multiple perspectives. As Lyons et al. point out, it is essential to examine the perspectives of multiple individuals: the "single-provider focus does not well represent clinical reality as experienced by interdisciplinary teams [22]."

Finally, we learned that improving care within the VA healthcare system (and perhaps other large healthcare organizations) can require high-level organizational involvement. For example, implementing wellness groups or clozapine clinics required active involvement from nutrition and pharmacy, respectively, which were medical center-wide services. Indeed, sometimes management of these services resided at the level of the Veterans Integrated Service Network (VISN; the 21 VA regions of the United States). Nutrition and pharmacy services did not respond to requests from staff at the level of mental health clinics, and this lack of responsiveness impeded our ability to implement a clozapine clinic or to involve the nutrition department in the wellness programs.

### EQUIP-2 Methods

This section describes: 1) the EQUIP-2 project and conceptual framework, 2) the evolution of the EQUIP-2 implementation strategy (i.e., interventions and tools), and 3) the formative evaluation component of EQUIP-2.

### EQUIP-2 overview

As noted above, the next phase of work building toward national roll-out of the EQUIP intervention is EQUIP-2 – a Step 4, Phase 2 multi-site evaluation (See Table 1). As the Overview to the *Series *notes [1], projects within this phase are considered "clinical trials to further refine and evaluate an improvement/implementation program." These trials involve a small sample of facilities conducting the implementation program under somewhat idealized conditions. Moreover, it is noted [4] that these projects require active research team support and involvement, plus modest real-time refinements to maximize the likelihood of success and to study the process for replication requirements. They employ formative evaluation (to monitor and feed back information regarding implementation and acceptance and impacts), as well as development and use of formal measurement tools and evaluation methods.

EQUIP-2 is a three-year project that was funded in January 2006, and aimed at our implementation strategy refinement and broad formative evaluation in eight sites across four VISNs that used the implementation approaches adopted by QUERI. As noted above, EQUIP-2 was funded as an SDP [4], which involves a unique set of expectations in terms of addressing what are called "quality gaps" (i.e., the current lack of evidence-based care for schizophrenia, described above).

The project reflects the growth in knowledge, both at the researcher and study reviewer levels, regarding implementation science. More specifically, unlike EQUIP, this study includes a conceptually-driven study of the process of implementation that includes the effect of various interventions on patients, clinicians, and organizations, and a more conceptually-based implementation strategy. The early implementation efforts described above also prompted the EQUIP-2 investigators to incorporate and/or strengthen several components of the multi-phasic evaluation as described by Stetler and colleagues [2]. These authors recommend: diagnostic analysis of organizational readiness (e.g., using relevant surveys) and interviews regarding attitudes and beliefs; implementation-focused evaluation examining the context where change is taking place; maintenance and optimization of research implementation interventions; and provision of feedback, e.g., regarding progress on targeted goals. They also recommend collecting data from experts, representative clinicians/administrators, and other key informants regarding both pre-implementation barriers and facilitators and post-implementation perceptions of the evidence-based practice and implementation strategy. All of these elements are being utilized in EQUIP-2 and are described further below with regard to the formative evaluation.

### Conceptual framework: Simpson Transfer Model & PRECEDE

Though informed by more than one conceptual framework, EQUIP-2 is organized around the Simpson Transfer Model (STM). This model guides the development and refinement of a diversified, flexible menu of tools and interventions to improve schizophrenia care. Incorporating the notion of readiness to change [23] at both the individual and organizational levels, Simpson developed a program change model for transferring research into practice [24]. The STM has provided important conceptual input to many studies in technology transfer [25-27]. This model involves four action stages: exposure, adoption, implementation, and practice. *Exposure *is dedicated to introducing and training in the new technology; *adoption *refers to an intention to try a new technology/innovation through a program leadership decision and subsequent support; *implementation *refers to exploratory use of the technology/innovation; and *practice *refers to routine use of the technology/innovation, likely with the help of customization of the technology/innovation at the local level. Crucial to moving from exposure to implementation are personal motivations of staff and resources provided by the institution (e.g., training, leadership), organizational characteristics such as "climate for change" (e.g., staff cohesion, presence of product champions, openness to change), staff attributes (e.g., adaptability, self-efficacy), and characteristics of the innovations themselves (e.g., complexity, benefit, observability).

EQUIP-2 also draws upon the PRECEDE planning model for designing behavior change initiatives [28]. Because the STM model does not recommend *specific *behavior change tools to be used in a knowledge transfer intervention, additional guidance is necessary regarding development of the implementation framework. The PRECEDE acronym stands for "predisposing, reinforcing, and enabling factors in diagnosis and evaluation." PRECEDE stresses the importance of applying multiple interventions to influence the adoption of targeted clinician behaviors. These include: 1) academic detailing and consultation with an opinion leader or clinical expert, which can help *predispose *clinicians to be willing and able to make the desired changes; 2) patient screening technologies, clinical reminders, and/or other clinical support tools that can *enable *clinicians to change; and 3) social or economic incentives that can *reinforce *clinicians' implementation of targeted behaviors.

A key part of the PRECEDE model is the active *participation *of the target audience in defining the issues and factors that influence targeted behaviors, and in developing and implementing solutions [28]. This participation principle is consistent with the *social marketing *framework, which emphasizes the importance of understanding a target audience's initial and ongoing perceptions of the innovation, in order to facilitate behavior change [29,30]. Both PRECEDE and social marketing theory state that messages and interventions should be tailored to perceptions in order to influence the desired behavior change.

Taken together, the models and frameworks discussed above suggest that the impact of implementation efforts will be maximized when they: 1) are based on assessments of the needs, barriers, and incentives of targeted end users; 2) are based on an understanding of the local context; 3) involve representatives of diverse stake-holder groups in the planning process; 4) use expert involvement in planning, especially when behaviors to be adopted and/or changed are complex; 5) draw on marketing principles for developing and disseminating intervention tools; and 6) secure support and involvement from top level management and product champions [31-33]. Each of these factors is integrated into the STM, which guides the EQUIP-2 strategy and formative evaluation. Table 5 provides an overview of how we will engage in each phase of the STM.

**Table 5:** Simpson Transfer Model stages and corresponding activities

STM stages	Intervention components and tools	Formative evaluation
Exposure	• Secure commitment	Developmental evaluation
	• Training and observation of care model by site PIs and regional project managers	• Organizational Readiness for Change (ORC: prior to implementation)
	• Review evidence	• Key informant interviews
	• Address values	
	• Identify and prioritize needs and treatment targets	
	• Begin tailoring care practice protocols	
	• Kick-off meeting and video conferences on treatments to be implemented	

Adoption	**Predisposing activities**:	Developmental evaluation
	• VISN Implementation Teams	Rogers' adoption questions:
	• Product champions	• Complexity
	• Continue tailoring care practice protocols	• Relative advantage
	• Continue to secure commitment, address values	• Observability

Implementation	**Enabling activities**:	Progress-focused evaluation
	• Patient self- assessment informatics (PAS) with provision of data to clinicians.	• PAS tracking (ongoing)
	• Treatment-specific implementation activities, such as help with wellness groups and liaison with supported employment.	Implementation-focused evaluation
	• Discuss and start using provider supports and incentives.	• Project documents (minutes from Implementation Team meetings, project managers' field notes, quality coordinators' logs: all ongoing).
		• Provider and clinic manager interviews (mid-implementation)

Practice	**Reinforcing activities (performance monitoring & feedback)**:	Interpretive evaluation
	• Monthly quality meeting and Quality Reports	• Provider & clinic manager interviews (post-implementation)
	• Quarterly conference calls re: treatment target implementation and use	• Computer system usability questionnaire
	• Implementation team meetings	
	• Continue tailoring with provider input	
	• Finalize provider supports and incentives	
	• Continue tailoring with leader input	

Sustainability	• Stakeholder feedback discussions	Interpretive evaluation
		• Level of Institutionalization
		• ORC

### Evolution of the schizophrenia implementation strategy

Several modifications were made in EQUIP-2 as a result of the findings and observations in EQUIP. An overview of each type of strategy is provided below; Table 4 (right column) notes the specific changes made in EQUIP-2 based on findings from EQUIP.

### Evidence-based clinical/therapeutic practices

EQUIP-2 is more targeted than EQUIP in its approach to strengthening specific evidence-based practices within the care model. EQUIP-2 focuses on quality improvement by assisting staff to implement specific evidence-based practices that have shown strong impacts on outcomes [7]. In addition, since EQUIP's onset, the VA has made a national commitment to implementing "recovery-oriented" practices in schizophrenia, which is embodied in the President's New Freedom Commission on Mental Health that was established in 2002 [34], and the VA's Mental Health Strategic Plan [10]. Thus, EQUIP-2 provides implementation support on evidence-based practices that support recovery. Each VISN involved was asked to choose two evidence-based practices from a list of four practices that EQUIP-2 was prepared to support (Table 6). All four VISNs chose the same two targets – wellness and supported employment.

**Table 6:** Evidence-based clinical/therapeutic practices that could be supported in EQUIP-2:

1. Clozapine for patients with severe psychosis, with the goal of increasing the proportion who receive clozapine (Evidence level^1 ^= 1b, [39]);
2. Wellness intervention for elevated weight, with the goals of increasing the proportion of patients receiving antipsychotic medication with less weight gain potential (Evidence level = 1a, [40]), and increasing the proportion of patients who receive a group-based wellness intervention [41]);
3. Family involvement to improve symptom control and functioning (Evidence level = 1a, [42]), with the goals of increasing the proportion of family members who are involved in developing the patient's treatment plan; and
4. Supported Employment for unemployment (Evidence level = 1b, [43]), with the goal of increasing the proportion of patients (who want to work) receiving evidence-based rehabilitation services that lead to competitive employment.

Evidence Pyramid

**1a: **Evidence obtained from meta-analysis of randomized controlled trials (RCTs)

**1b: **Evidence obtained from at least 1 RCT

**2a: **Evidence obtained from at least 1 well-designed controlled study without randomization

**2b: **Evidence obtained from at least 1 other type of well-designed quasi-experimental study

**3: **Evidence obtained from well-designed non-experimental descriptive studies, such as comparative studies, correlation studies and case control studies

**4: **Evidence obtained from expert committee reports or opinions and/or clinical experience of respected authorities

### Delivery system interventions

During the intervention period, there is a monthly quality meeting at each intervention clinic. This quality meeting is "local" and the site PI (principal investigator), quality coordinator, product champion, and clinicians attend this meeting. During the meeting, each clinician is given his/her personal "Quality Report." Quality meetings: 1) allow pervasive quality problems to be identified, 2) optimize teamwork by encouraging group problem-solving on patient management problems, and 3) identify resources needed to address care problems. Lastly, high-achieving clinicians are discussed (i.e., those who are accomplishing the specific goals of each care target) and incentives are distributed.

### Adoption/implementation tools

In terms of marketing, all of the sites had an explicit project "kick-off" that signalled the start of the project and promoted a sense of excitement. Educational activities and trainings commenced both at the coordinating center and at the individual clinics.

In order to promote further engagement and collaboration, additional levels of personnel are involved in the project from its inception. Prior to enrolment, we have had monthly planning calls involving clinic staff, regional managers, and medical center leadership. These calls address practical issues regarding study set-up, as well as plans for marketing. Once enrollment begins, we will have monthly Implementation Team calls involving site PIs, site project directors, product champions, VISN-level staff, and the research team. These calls will examine and address all implementation issues as they arise and will work toward sustainability of the model. During the course of implementation, we maintain the research nurse position from EQUIP in the form of "Quality Coordinators." These individuals were reported to make a difference in EQUIP, not only to clinicians, in that they provided additional clinical information about patients, but also to patients, in that they provided an additional source of support. Further, we engage case managers from the beginning of the project.

We encourage staff to identify who they go to for expertise in the chosen care targets, and ask that individual to volunteer as product champions for the project. We identify product champions based on this information and ask them to participate in monthly Implementation Team calls, as well as other mechanisms of involvement.

### Formative evaluation

As noted above, this Phase 2 implementation project involves a more formal evaluation component, due to the importance at this stage of program refinement. Below we describe each component of the formative evaluation.

### Developmental evaluation

In EQUIP, we observed that organizational climate and staff engagement and structure significantly affected the degree to which the tools presented in the project were effective. This observation is consistent with emerging implementation science, which itself is increasingly recognizing the importance of context. In order to better understand and "diagnose" [2] the organizational climate of the sites, the Simpson Transfer Model organizational readiness measures will be used in EQUIP-2. We also conduct key informant interviews in order to better understand the clinics' preparedness for the intervention.

### Implementation-focused evaluation

Each month during implementation, there are Implementation Team meetings, which serve to link intervention sites and the research team from the coordinating center. Here, barriers and facilitators to implementation are identified and discussed, and group problem-solving and any needed reorganization of care is planned and documented. Product champions and other site personnel also report on any informal feedback they have received about problems with the implementation. As implementation continues, this team works toward sustainability of the model. Minutes from these meetings, project managers' field notes, and quality coordinators' logs are analyzed to evaluate implementation throughout the intervention period. In addition, midway through the intervention, the research team conducts semi-structured interviews with clinicians and clinic managers to evaluate the operationalization of the intervention, necessary refinements to the intervention, and areas of desired guidance. In order to reduce burn-out, promote and maintain enthusiasm for the project, and to optimize successful implementation overall, various interventions are modified if feedback and other formative data indicate that change is necessary.

### Progress-focused evaluation

During the course of the project, in order to monitor progress toward the project's goals, we evaluate the degree to which physicians respond to the patient self-assessments. For example, do they provide the necessary and/or requested referrals to supported employment, and do they refer patients to wellness groups for weight management. We also assess the Quality Reports for other outcome progress. When we find that progress is not being made toward the goals, we work in coordination with the clinics to identify barriers to achieving the goals and strategies for addressing and mitigating the barriers.

### Interpretive evaluation

At the conclusion of the project, we will conduct semi-structured interviews with the clinicians and clinic managers regarding the usefulness of the EQUIP-2 strategy, their satisfaction with the implementation process, barriers to and facilitators of implementation, and recommendations for future refinements [2]. In order to re-evaluate the delivery system interventions, we will collect quantitative data about the usability of the informatics system. Measures of organizational readiness will be repeated, in order to describe changes in organizational climate during the course of the project, as another potential influence on successful implementation. And the extent to which the care model has become "institutionalized," (i.e., degree to which the care model has become part of routine clinical practice) will be examined.

For the final interpretive evaluation, we will explore all formative evaluation data in light of our outcome data in order to provide: alternative explanations of results; clarification of our implementation effort success (or failure); and assessment of the potential for reproducibility of our implementation strategy in a broader segment of the VA [4].

## Discussion

The evolution of the EQUIP implementation program took shape during the development of the field of implementation science. The experience in EQUIP, combined with the guidance received from the subsequent EQUIP-2 protocol, led to a Step 4, Phase 2 activity that more explicitly engages in evidence-based quality improvement and in formal evaluation. Although the formative evaluation in EQUIP was more limited compared to recently developed formative evaluations, it produced important new information regarding quality improvement in schizophrenia. It has been widely acknowledged that there are major problems with the quality of routine care for schizophrenia, but there has been limited research on how to improve this care and on the challenges to improving care. EQUIP identified effective and ineffective methods and strategies for improving care, and provided results that can be of substantial use to people working to improve treatment and outcomes in this disorder.

We agree with Kitson, Harvey, & McCormack [35] that the level and nature of evidence, the environment in which research is placed, and the method in which the process of implementation is undertaken can be equally important in successful implementation: "Implementation may not be successful within a context that is receptive to change, because there is non-existent or ineffective facilitation ... For implementation to be successful, there needs to be a clear understanding of the nature of evidence being used, the quality of context in terms of its ability to cope with change and type of facilitation needed to ensure a successful change process" (p. 152). Accordingly, our approach in EQUIP-2 addresses the interventions, environment, and process equally, and involves thorough assessment of each component.

Clearly a multi-faceted evaluation is needed to develop a comprehensive understanding of barriers to and facilitators of implementation of the chronic illness care model in schizophrenia. In this disorder, barriers to improving care in EQUIP varied by evidence-based practice, and included under-developed clinician competencies, burn-out among clinicians, limited availability of psychosocial treatments, inadequate attention to medication side-effects, and organization of care that was not consistent with high quality practice. Facilitators to improving care included interest among clinicians and policymakers in improving care, and robust specialty mental health services. Summative evaluation is not sufficient to understand these components. Instead, as begun in EQUIP and more fully developed in EQUIP-2, we believe that a conceptually-driven formative evaluation can provide more detail as to the interactions between interventions, process, and context. Research such as EQUIP-2 should help to determine the relative importance of each component, providing direction as to when one component needs more attention than another during the course of a quality improvement implementation project [36]. Scientifically-based qualitative evaluations of quality improvement in schizophrenia may guide project development, strengthen future stages of intervention development (as illustrated in the development of EQUIP-2), and inform future mixed methods evaluation within the field of implementation science.

## Competing interests

The author(s) declare that they have no competing interests.

## Authors' contributions

AHB conducted the independent qualitative study, analyzed the data, and drafted the manuscript. ANC served as the project director, and she conducted the pre-post EQUIP semi-structured interviews, analyzed the data, and helped draft the manuscript. MJC collaborated on instrument development and analyses, and helped draft the manuscript. CK served as a product champion for the project, and helped draft the manuscript. ASY conceived of the study, participated in its design and coordination, and helped draft the manuscript. All authors read and approved the final manuscript.

## Disclaimer

The findings and conclusions in this document are those of the authors, who are responsible for its contents, and do not necessarily represent the views of the U.S. Department of Veterans Affairs.
